# Mechanisms of Paradoxical Activation of AMPK by the Kinase Inhibitors SU6656 and Sorafenib

**DOI:** 10.1016/j.chembiol.2017.05.021

**Published:** 2017-07-20

**Authors:** Fiona A. Ross, Simon A. Hawley, F. Romana Auciello, Graeme J. Gowans, Abdelmadjid Atrih, Douglas J. Lamont, D. Grahame Hardie

**Affiliations:** 1 Division of Cell Signalling & Immunology, College of Life Sciences, University of Dundee, Dow Street, Dundee DD1 5EH, UK; 2 Fingerprints Proteomics Facility, School of Life Sciences, University of Dundee, Dundee DD1 5EH, UK

**Keywords:** AMPK, AMP-activated protein kinase, SU6656, sorafenib, MRT199665, kinase inhibitors

## Abstract

SU6656, a Src kinase inhibitor, was reported to increase fat oxidation and reduce body weight in mice, with proposed mechanisms involving AMP-activated protein kinase (AMPK) activation via inhibition of phosphorylation of either LKB1 or AMPK by the Src kinase, Fyn. However, we report that AMPK activation by SU6656 is independent of Src kinases or tyrosine phosphorylation of LKB1 or AMPK and is not due to decreased cellular energy status or binding at the ADaM site on AMPK. SU6656 is a potent AMPK inhibitor, yet binding at the catalytic site paradoxically promotes phosphorylation of Thr172 by LKB1. This would enhance phosphorylation of downstream targets provided the lifetime of Thr172 phosphorylation was sufficient to allow dissociation of the inhibitor and subsequent catalysis prior to its dephosphorylation. By contrast, sorafenib, a kinase inhibitor in clinical use, activates AMPK indirectly by inhibiting mitochondrial metabolism and increasing cellular AMP:ADP and/or ADP:ATP ratios.

## Highlights

• SU6656 activates AMPK independently of the Src kinases Src, Yes, and Fyn
• SU6656 acutely inhibits AMPK by competing with ATP at the active site
• SU6656 paradoxically activates AMPK by enhancing phosphorylation by LKB1
• By contrast, sorafenib activates AMPK indirectly by increasing cellular AMP/ADP

## Graphical Abstract

**Figure:**
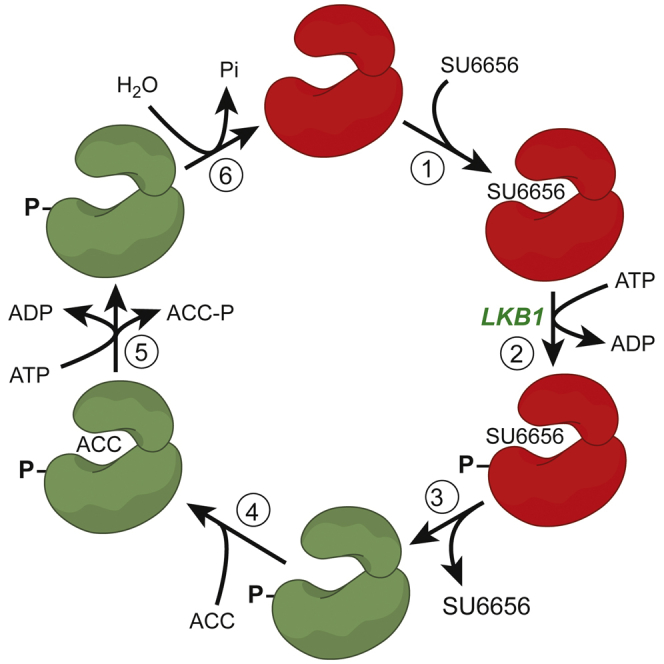

## Teaser

The kinase inhibitors SU6656 and sorafenib paradoxically activate AMPK. Ross et al. show that SU6656 inhibits AMPK by binding directly at the catalytic site, yet promotes phosphorylation and activation by LKB1, while sorafenib activates AMPK indirectly by inhibiting mitochondrial function.

## Introduction

AMP-activated protein kinase (AMPK) is a sensor of cellular energy status. It exists in eukaryotes as heterotrimeric complexes comprising catalytic α and regulatory β and γ subunits (Hardie et al., 2016, Ross et al., 2016b), occurring in mammals as multiple isoforms (α1/α2; β1/β2; γ1/γ1/γ3) (Stapleton et al., 1996, Thornton et al., 1998, Cheung et al., 2000). AMPK can be activated >100-fold by phosphorylation at a conserved threonine (Thr172) within the activation loop of the kinase domain. The major upstream kinase phosphorylating Thr172 is a complex containing LKB1, which also phosphorylates and activates a family of 12 AMPK-related kinases; Thr172 can also be phosphorylated by the Ca^2+^-activated kinase, CaMKK2 (Hardie et al., 2016, Ross et al., 2016b). The AMPK-β subunits contain a carbohydrate-binding module (β-CBM), and a cleft between this domain and the small lobe of the α subunit kinase domain forms the allosteric drug and metabolite (ADaM) site (Langendorf and Kemp, 2015), where activators such as the natural product, salicylate, and the synthetic compounds, 991 and A769662, bind (Hawley et al., 2012, Xiao et al., 2013). The γ subunits contain up to three sites at which the regulatory nucleotides AMP, ADP, and ATP can bind in competition (Xiao et al., 2007, Chen et al., 2012). Binding of AMP or ADP promotes net Thr172 phosphorylation by enhancing phosphorylation and inhibiting dephosphorylation (Oakhill et al., 2011, Xiao et al., 2011), while binding of AMP causes further allosteric activation, at least with γ1 and γ2 complexes (Ross et al., 2016a). AMPK is therefore activated by stresses that interfere with ATP synthesis or accelerate ATP consumption, causing increases in cellular AMP:ATP and ADP:ATP ratios. It acts to restore cellular energy balance by switching on catabolic pathways that generate ATP, while switching off ATP-consuming processes.

Most studies suggest that LKB1 complexes are constitutively active, with Thr172 phosphorylation being regulated instead by binding of adenine nucleotides to AMPK (Hardie et al., 2016, Ross et al., 2016b). However, it has been proposed that LKB1 is regulated by tyrosine phosphorylation by members of the Src kinase family. Following observations of metabolic changes in Fyn knockout mice (Bastie et al., 2007), treatment of mice with the Src kinase inhibitor SU6656 increased fatty acid oxidation associated with increased phosphorylation of AMPK and its downstream target acetyl-CoA carboxylase (ACC) in skeletal muscle (Yamada et al., 2010). Treatment of C2C12 cells with SU6656 caused translocation of GFP-LKB1 from nucleus to cytoplasm, while overexpression of Fyn had the opposite effect. Finally, Fyn was found to phosphorylate LKB1 on Tyr261 and Tyr365, leading to a model in which LKB1 tyrosine phosphorylation causes nuclear retention, reducing its ability to activate cytoplasmic AMPK. Another group reported that the Src kinase Lck phosphorylated LKB1 on Tyr36, as well as Tyr261/365 (Cao et al., 2011). More recently, Yamada et al. (2016) proposed a different mechanism in which Fyn phosphorylates Tyr436 on the AMPK-α subunit, causing its direct inhibition.

We now confirm that SU6656 activates AMPK but show that it does this not by modulating tyrosine phosphorylation of LKB1 or AMPK but, surprisingly, by direct interaction with the AMPK kinase domain. Recently sorafenib, another kinase inhibitor already licensed for treatment of some cancers (Gadaleta-Caldarola et al., 2015), was reported to activate AMPK in tumor cell lines (Fumarola et al., 2013, Groenendijk et al., 2015). We show that, in contrast to SU6656, sorafenib activates AMPK indirectly by inhibiting mitochondrial metabolism and increasing cellular AMP:ATP and ADP:ATP ratios.

## Results

### SU6656 Activates AMPK but Not AMPK-Related Kinases

**Figure 1:**
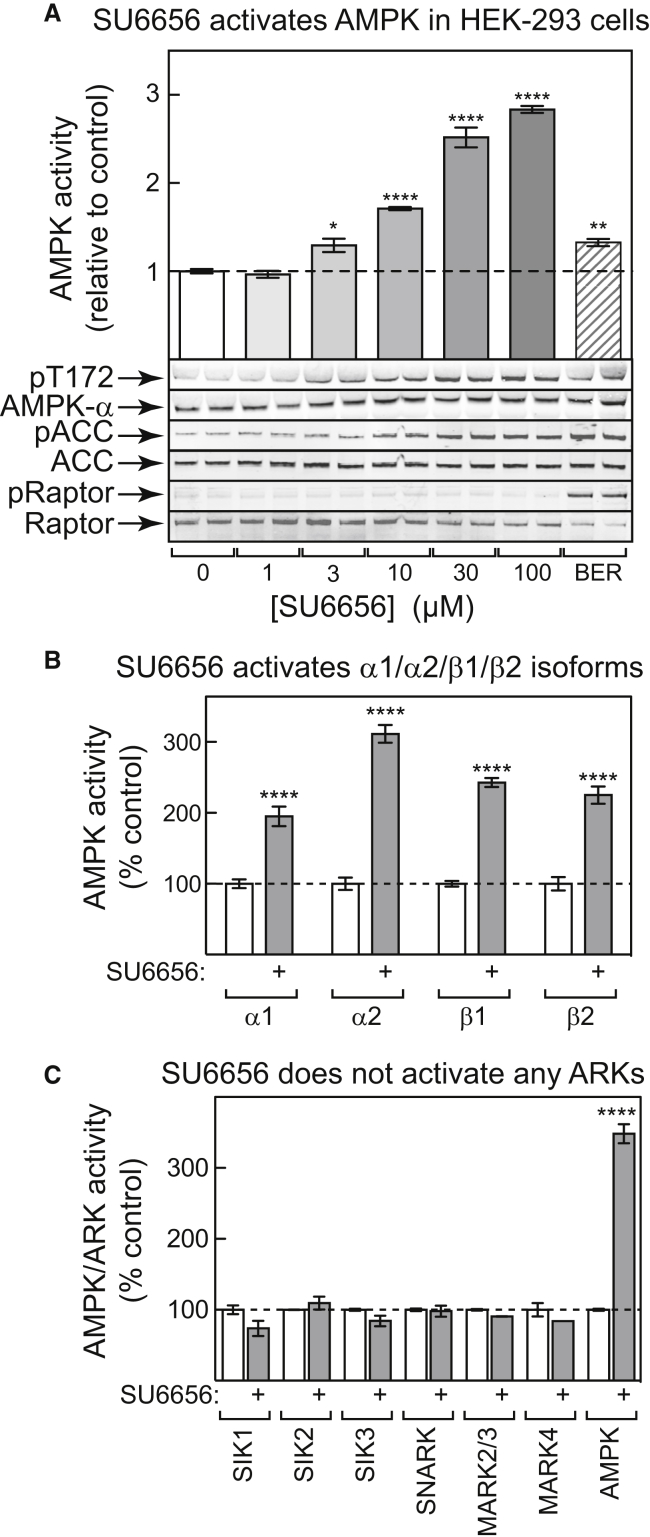
SU6656 Activates AMPK but Not AMPK-Related Kinases (A) HEK293 cells were incubated with the indicated concentrations of SU6656 or berberine (300 μM) for 1 hr; total AMPK activity was assayed in immunoprecipitates (top; mean ± SEM, n = 4), and phosphorylation of AMPK (Thr172), ACC (Ser80), and Raptor (Ser792) analyzed by western blotting (bottom; n = 2). (B) HEK293 cells were incubated with 100 μM SU6656 (1 hr), AMPK complexes containing α1, α2, β1, and β2 immunoprecipitated using isoform-specific antibodies, and AMPK activity measured (mean ± SEM, n = 4). (C) HeLa cells stably expressing LKB1 (Fogarty et al., 2010) were incubated with 100 μM SU6656 for 1 hr, AMPK or individual ARKs immunoprecipitated, and kinase assays carried out (antibodies that distinguish MARK2/MARK3 were not available, so they were assayed together). Results are means ± SEM (n = 2). In all panels, asterisks indicate significant differences from controls without SU6656. *p < 0.05, **p < 0.01, ****p < 0.0001.

Treatment of HEK293 cells with increasing concentrations of SU6656 caused activation of AMPK that correlated with Thr172 phosphorylation; it also increased phosphorylation of one downstream target, ACC, but not another, Raptor. Another AMPK activator, berberine, caused a much smaller degree of AMPK activation and Thr172 phosphorylation, yet had similar effects on ACC phosphorylation and did promote Raptor phosphorylation (Figure 1A). By immunoprecipitating with isoform-specific antibodies, SU6656 was found to activate AMPK complexes containing the α1, α2, β1, and β2 subunit isoforms (Figure 1B). In HEK293 cells, AMPK occurs primarily as γ1 complexes (Hawley et al., 2010), but we show later (Figure 3A) that γ2 complexes can also be activated by SU6656.

If SU6656 acts by blocking phosphorylation of LKB1 by Fyn and promoting migration of LKB1 to the cytoplasm (Yamada et al., 2010), the AMPK-related kinases (ARKs) downstream of LKB1 should also be activated. All of the ARKs except BRSK1 and BRSK2 are expressed in the LKB1-null HeLa cell line, although they are only active if LKB1 is re-expressed (Lizcano et al., 2004). While SU6656 robustly activated AMPK in HeLa cells expressing LKB1, none of the ARKs assayed were activated (Figure 1C).

### AMPK Activation Does Not Require Src Kinases or LKB1 Tyrosine Phosphorylation

**Figure 2:**
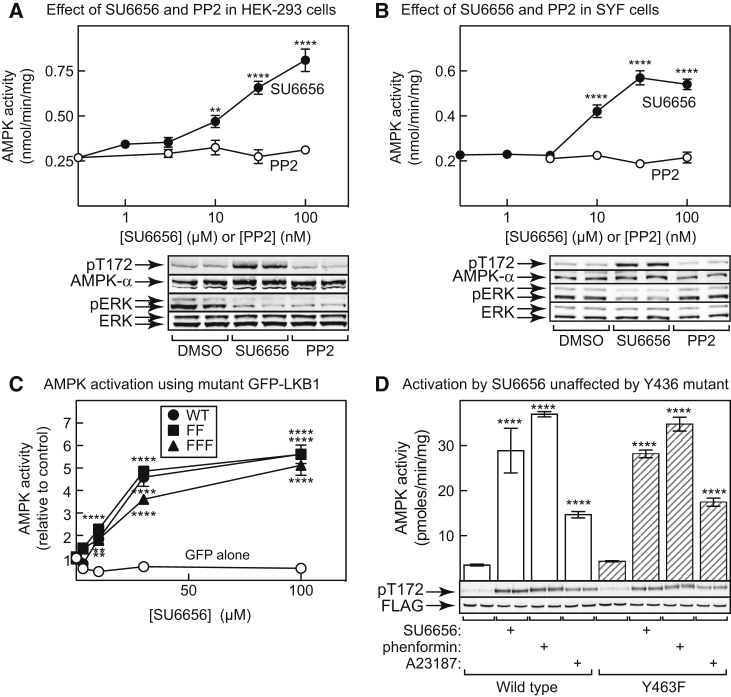
AMPK Activation by SU6656 Does Not Require an Src Family Kinase or Tyrosine Phosphorylation of Either LKB1 or AMPK (A) HEK293 cells were incubated with the indicated concentrations of SU6656 or PP2 for 1 hr and AMPK activity assayed. Top: AMPK activity (mean ± SEM, n = 4); bottom: western blots showing phosphorylation of Thr172 on AMPK, and of ERK1/2, in cells treated with DMSO (control), SU6656 (100 μM), or PP2 (100 nM) (n = 2). (B) As (A), but in SYF cells. (C) HeLa cells were transfected with DNAs encoding GFP alone, or GFP fused to WT, Y261F/Y365F (FF), or Y36F/Y261F/Y365F (FFF) mutants of LKB1; cells were treated with increasing concentrations of SU6656 for 1 hr and AMPK activity determined (mean ± SEM, n = 4). See also Figure S1. (D) HEK293 cells were transfected with DNAs encoding FLAG-tagged AMPK-α2 with or without a Y436F mutation. Cells were incubated with DMSO (control), 100 μM SU6656, 10 mM phenformin, or 10 μM A23187 for 1 hr; AMPK complexes were immunoprecipitated using anti-FLAG antibody and assayed. The top panels show kinase activities (mean ± SD, n = 3); the bottom panels show blots from the same experiment (n = 2). In all panels, asterisks indicate significant differences from DMSO controls; in (D), there were no significant differences between the WT and Y463F mutant. **p < 0.01, ****p < 0.0001.

The results in Figure 1C provided the first hint that AMPK activation by SU6656 might not be working via the previously suggested mechanism involving LKB1 phosphorylation by Fyn. Consistent with this, PP2, a more selective inhibitor of Src kinases than SU6656 (Bain et al., 2007) although not specific for them (Brandvold et al., 2012), had no effect on AMPK activity or Thr172 phosphorylation in HEK293 cells. PP2 and SU6656 both reduced phosphorylation of Erk1/Erk2, as expected for Src inhibitors (Figure 2A). We next tested the effect of SU6656 in SYF cells, which are mouse embryo fibroblasts (MEFs) with null alleles for Src, Yes, and Fyn, the three Src kinases expressed in MEFs (Klinghoffer et al., 1999). PP2 still had no effect on AMPK, but now also had no effect on Erk1/2, as expected. However, SU6656 still activated and increased Thr172 phosphorylation of AMPK (Figure 2B). Surprisingly, it also inhibited Erk1/2 phosphorylation, suggesting additional Src kinase-independent effects.

We next transiently transfected DNAs encoding GFP, or GFP-tagged wild-type (WT), Y261F/Y365F (FF), or Y36F/Y261F/Y365F (FFF) mutants of LKB1 into HeLa cells, which lack endogenous LKB1. AMPK was activated (Figure 2C) and phosphorylated on Thr172 (Figure S1, Supplemental Information) by SU6656 treatment equally in cells expressing the WT, FF, or FFF mutants, although not with the GFP control.

To test whether AMPK activation by SU6656 was due to inhibition of phosphorylation of Tyr436 on the AMPK-α subunits by Fyn (Yamada et al., 2016), we expressed FLAG-tagged WT or Y436F mutant human AMPK-α2 in HEK293 cells, treated with various activators and measured AMPK activity in anti-FLAG immunoprecipitates. SU6656, phenformin (which acts by inhibiting mitochondrial complex I and increasing AMP/ADP) and A23187 (an ionophore that increases intracellular Ca^2+^ and activates CaMKK2) all activated AMPK, and these responses were unaffected by the Y436F mutation (Figure 2D).

### Sorafenib, but Not SU6656, Activates AMPK by an AMP-Dependent Mechanism

**Figure 3:**
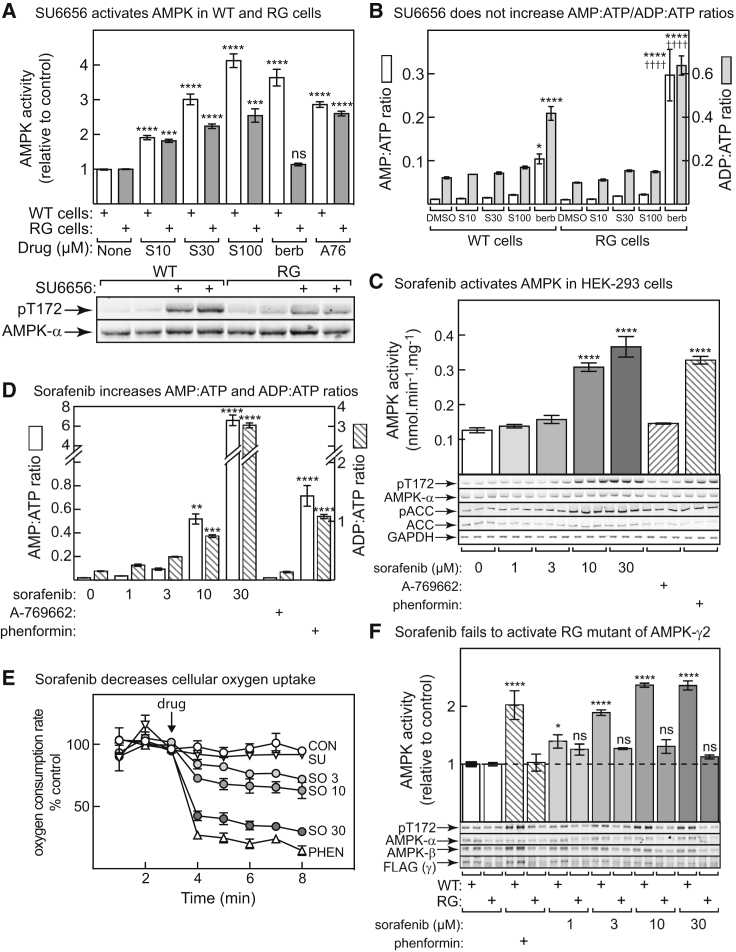
Sorafenib, but Not SU6656, Activates AMPK by Inhibiting Mitochondrial Metabolism and Increasing Cellular AMP (A) Top: HEK293 cells expressing wild-type (WT) AMPK-γ2 or the R531G (RG) mutant were treated for 1 hr with SU6656 at 10, 30 or 100 μM (S10, S30, S100), 300 μM berberine (berb) or 300 μM A769662 (A76) and AMPK assayed (mean ± SEM, n = 4); bottom: blots of Thr172 phosphorylation and total AMPK-α in cells (n = 2) incubated with DMSO or 100 μM SU6656 for 1 hr. (B) WT or RG cells were treated as in (A) and nucleotides extracted for analysis (mean ± SD, n = 3). (C) Effects of incubation (1 hr) of WT HEK293 cells in increasing concentrations of sorafenib, A769662 (300 μM) or phenformin (10 mM) on AMPK activity (top, mean ± SEM, n = 3) and on phosphorylation of Thr172 and ACC (bottom). (D) Effects of incubation of WT HEK293 cells (1 hr) in increasing concentrations of sorafenib, A769662 (300 μM) or phenformin (10 mM) on cellular AMP:ATP/ADP:ATP ratios (mean ± SD, n = 3). (E) (See also Figure S2) Time courses of cellular oxygen uptake before and after addition of the indicated concentrations of sorafenib (SO, μM), SU6656 (100 μM) or phenformin (PHEN, 10 mM) (mean ± SEM, n = 4). (F) Effect of various concentrations of sorafenib or phenformin (10 mM) on activation (top, mean ± SEM, n = 3) or phosphorylation (bottom) of AMPK in cells expressing wild-type AMPK-γ2 (WT) or the R531G mutant AMPK-γ2 (RG). In all panels, asterisks show significant differences from DMSO controls (ns, not significant). In (B), daggers (†) show significant differences between results in WT and RG cells. *p < 0.05, **p < 0.01, ***p < 0.001, ****p < 0.0001, ^††††^p < 0.0001.

The next possibility was that, like several other AMPK-activating drugs, SU6656 inhibits mitochondrial ATP synthesis, and thus activates AMPK indirectly by increasing cellular AMP/ADP. To test this, we used cell lines inducibly expressing either WT AMPK-γ2 or an AMP/ADP-resistant R531G (RG) mutant (Hawley et al., 2010). As expected, berberine (an inhibitor of mitochondrial complex I) activated AMPK in the WT but not the RG cells, while A769662 (which directly binds AMPK at the ADaM site; Xiao et al., 2013), activated AMPK in both. SU6656 behaved like A769662 in that it activated AMPK and enhanced Thr172 phosphorylation in both WT and RG cells, although the effects were somewhat smaller in the latter (Figure 3A). There were no significant effects of SU6656 on cellular AMP:ATP or ADP:ATP ratios in WT or RG cells, although they were robustly increased by berberine, to a greater extent in RG than in WT cells (Figure 3B). There were no effects of SU6656 on cellular oxygen uptake, unlike berberine. Subsequent addition of the uncoupler 2,4-dinitrophenol (DNP), which prevents the respiratory chain from being limited by ADP supply, increased oxygen uptake identically in control and SU6656-treated cells but had no effect following berberine, as expected for a complex I inhibitor (Figure S2).

We also examined the effects of sorafenib, another kinase inhibitor reported to paradoxically activate AMPK (Fumarola et al., 2013, Groenendijk et al., 2015). Sorafenib activated AMPK and promoted Thr172 and ACC phosphorylation in HEK293 cells (Figure 3C), with significant effects at 10 and 30 μM (peak plasma sorafenib in human clinical trials was 6–20 μM; Strumberg et al., 2007). Sorafenib also increased cellular AMP:ATP and ADP:ATP ratios to a similar or even greater extent than phenformin (Figure 3D). Moreover, like phenformin (but unlike SU6656), it rapidly inhibited cellular oxygen uptake at concentrations from 3 to 30 μM (Figure 3E). We also examined its effects in cells expressing the AMPK-γ2 RG mutant, generated by transient transfection. Neither AMPK activity nor Thr172 phosphorylation were significantly elevated by sorafenib in the RG cells, unlike the WT cells (Figure 3F).

### SU6656 Does Not Activate AMPK by Binding at the ADaM Site

**Figure 4:**
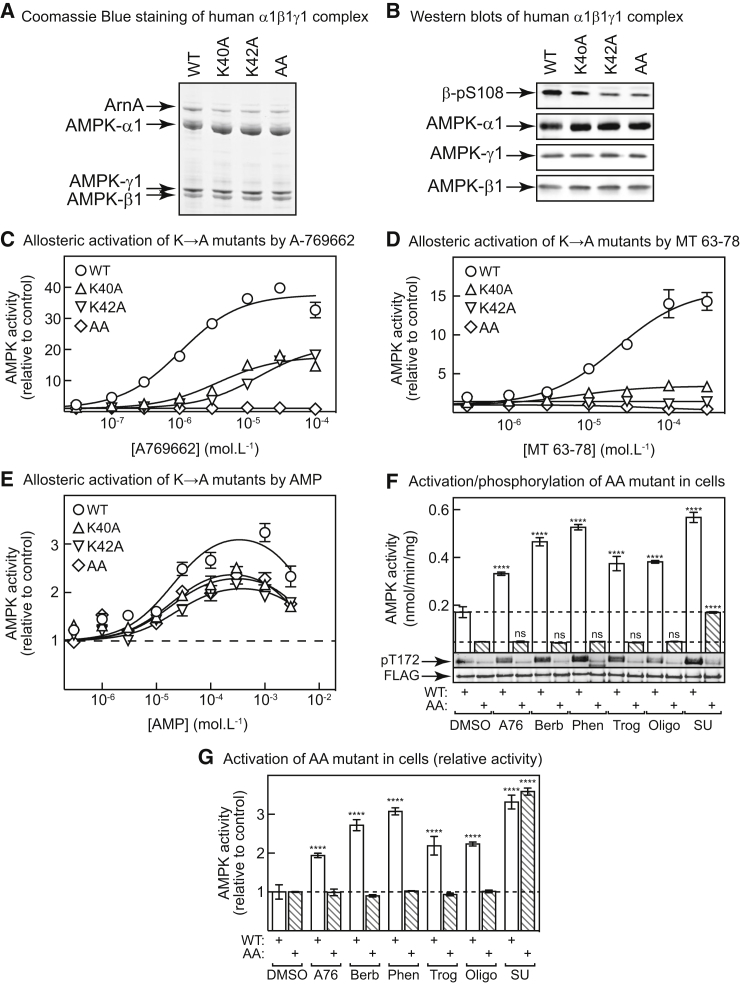
MT 63-78, but Not SU6656, Activates AMPK by Binding at the ADaM Site (A) Coomassie blue-stained gel of purified, bacterially expressed human α1β1γ1 complex, WT or with K40A, K42A, or K40A/K42A (AA) mutations in α1; ArnA is a contaminating protein from *Escherichia coli* identified by peptide mass fingerprinting. (B) Western blotting of the same preparations as in (A). (C) Allosteric activation of WT and mutant α1β1γ1 complexes (phosphorylated on β1-Ser108 but not α1-Thr172) by A769662. Data are expressed relative to the basal activity in the absence of activator and were fitted to the equation: Y = 1 + ((Activation − 1) × X)/(EC_50_ + X), where Y is activity, X is activator concentration, Activation is the maximal activation and EC_50_ is the concentration giving half-maximal activation. Parameters for the WT are quoted in the main text, the Activation and EC_50_ values for the K40A, K42A, and AA mutants were 18 ± 0.7-fold, 21 ± 0.6-fold, and 1.0 ± 0.03-fold, and 4 ± 0.7, 14 ± 0.6, and 0.001 ± 0.002 μM, respectively; continuous lines are theoretical curves drawn using these parameters. (D) Allosteric activation of WT and mutant α1β1γ1 complexes by MT 63-78, curve fitting as for (C). Parameters for the WT are quoted in the main text, the Activation and EC_50_ for the K40A mutant were 3.4 ± 0.1-fold and 7 ± 2 μM; fitting for the K42A and AA mutants did not yield sensible values. (E) Allosteric activation of WT and mutant α1β1γ1 complexes by AMP. Data were fitted to the equation for activation/inactivation by AMP (Gowans et al., 2013). Best-fit values for activation and EC_50_ are given in the main text; values for IC_50_ were 8.5 ± 4.1, 6.1 ± 1.9, 11.9 ± 3.8, and 8.8 ± 4.6 mM (WT, K40A, K42A, and AA); continuous lines are theoretical curves drawn using these parameters. (F) Activation of WT and AA mutant by various AMPK activators in HEK293 cells. Cells were transfected with DNAs encoding FLAG-tagged AMPK-α1 (WT or AA mutant) and treated with A769662 (300 μM), berberine (300 μM), phenformin (10 mM), troglitazone (100 μM), oligomycin (1 μM), or SU6656 (100 μM) for 1 hr. FLAG-tagged complexes were isolated by immunoprecipitation and AMPK activity determined (mean ± SEM, n = 2). Asterisks indicate significant differences from DMSO controls. The bottom panel shows western blotting of the anti-FLAG precipitates. ****p < 0.0001; ns, not significant. (G) Same experiment as (F), but results expressed relative to DMSO controls. ****p < 0.0001.

We next tested whether SU6656 activates AMPK by binding at the ADaM site. Another recently described activator, MT 63-78, may bind this site since, like A769662 and salicylate, it is a more potent activator of AMPK complexes containing β1 rather than β2 (Zadra et al., 2014). In crystal structures, Lys40 and Lys42 in α1 (Lys29/Lys31 in α2) are either directly involved in interactions with the activating ligand in the ADaM site or form electrostatic interactions with the autophosphorylated side chain of Ser108 in AMPK-β1, thus stabilizing the site; mutation of both lysines abolished activation by A769662 (Xiao et al., 2013, Li et al., 2015). We therefore expressed WT or single (K40A, K42A) or double (K40A/K42A, AA) α1 mutants in the context of the human α1β1γ1 complex (Figure 4A). Bacterially expressed AMPK complexes are not phosphorylated on Thr172 but can be allosterically activated by A769662 as long as β1-Ser108 is autophosphorylated (Scott et al., 2014a), which was the case with the WT and all mutants (Figure 4B). As expected, A769662 caused a large allosteric activation of the WT complex (activation = 38 ± 1-fold, concentration causing half-maximal effect [EC_50_] = 1.0 ± 0.1 μM [±SEM]). The single K40A or K42A mutations exhibited greatly reduced activation by A769662, while the double (AA) mutation abolished it (Figure 4C). Similar results were obtained with MT 63-78 (WT: activation = 16 ± 1-fold, EC_50_ = 23 ± 4 μM) (Figure 4D). By contrast, AMP still allosterically activated both the single and double mutants (Figure 4E). Although maximal activation was slightly reduced by the mutations (3.3 ± 0.3, 2.6 ± 0.1, 2.2 ± 0.1, and 2.4 ± 0.2-fold activation for WT, K40A, K42A, and AA mutant, respectively), there were no significant effects on the EC_50_ for AMP (20 ± 7, 19 ± 6, 23 ± 6, and 18 ± 8 μM).

We next expressed the FLAG-tagged WT or AA mutant of AMPK-α1 by transient transfection in HEK293 cells, treated with various agents, and measured AMPK activity in anti-FLAG immunoprecipitates. In Figure 4F, the results are expressed as absolute activities and are accompanied by blots showing Thr172 phosphorylation. For reasons that remain unclear, the AA mutation caused a 3- to 4-fold drop in kinase activity and Thr172 phosphorylation in the DMSO control, which is why the activities are also expressed relative to the DMSO control in Figure 4G. As expected, A769662, berberine, phenformin, troglitazone, oligomycin, and SU6656 activated AMPK and caused Thr172 phosphorylation with the WT complexes, and the AA mutation completely prevented the effect of A769662. More surprisingly, the effects of agents that increase cellular AMP:ATP, either by inhibiting the respiratory chain (berberine, phenformin, troglitazone) or the F1 ATP synthase (oligomycin), were also abolished by the AA mutation (note that any allosteric effects are lost during immunoprecipitation; any effects remaining are due to changes in Thr172 phosphorylation). However, SU6656 still caused a 3-fold increase in activity and Thr172 phosphorylation with both WT and AA mutant, despite the lower basal activity in the latter (Figure 4G), confirming that it acts by binding to site(s) distinct from either A769662 or AMP.

### SU6656 and AMP Promote Thr172 Phosphorylation by Binding to the Catalytic Site: Studies in Cell-Free Systems

**Figure 5:**
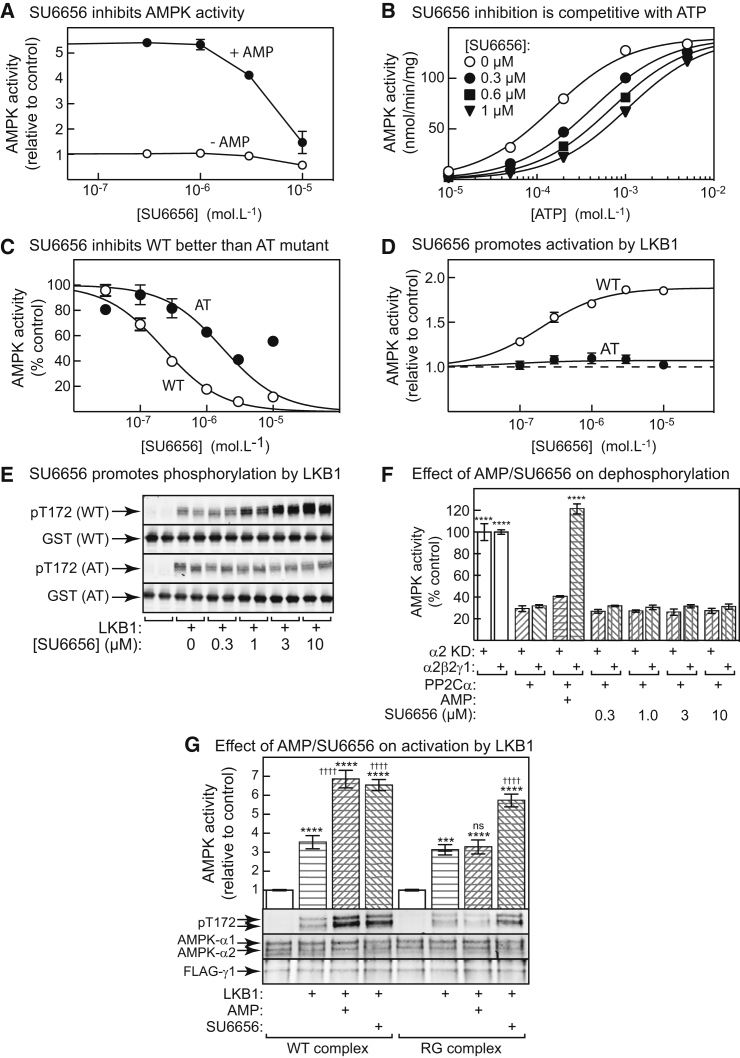
SU6656 Promotes AMPK Activation by Binding to the Catalytic Site: Studies in Cell-Free Assays (A) Effect of increasing concentrations of SU6656 ± 1 mM AMP in the presence of 2 mM ATP on the activity of purified rat liver AMPK. (B) Effect of ATP concentration on kinase activity of α2-KD in the presence of increasing concentrations of SU6656. Results (mean, n = 2) were fitted to the equation Y= (Vmax × X)/(Km × (1 + I/Ki) + X), where Y is the activity, X is the ATP concentration, I is the concentration of SU6656, and Ki is the inhibitory constant for SU6656. The curves were generated using this equation with the best-fit values of Vmax, Km, and Ki (shared between all four curves) quoted in the main text. (C) Inhibition of phosphorylated α2-KD (WT or AT mutant) by SU6656. Results (mean ± SEM, n = 2) were fitted to the equation Y = 100 − (100 × X/(IC_50_ + X)); curves were generated using this equation with values for IC_50_ quoted in the main text. The reduced inhibition at 10 μM SU6656 may be due to the inhibitor coming out of solution, so those data were not used for curve fitting. (D and E) Effect of increasing concentrations of SU6656 on activity (D) and Thr172 phosphorylation (E) induced by LKB1 with unphosphorylated α2-KD (WT/AT mutant). Results in (D) are mean ± SEM (n = 3); and were fitted to same equation as Figure 4C. Curves were generated using this equation with values for Activation and EC_50_ of WT quoted in the main text, and values of 1.07-fold and 0.07 μM for the AT mutant. See also Figure S3. (F) Effect of AMP (2 mM) and SU6656 on inactivation of AMPK by PP2Cα in the presence of 5 mM ATP. Results are mean ± SEM (n = 3); asterisks indicate results significantly different from controls with PP2Cα but without AMP or SU6656. (G) Effects of AMP or SU6656 on activation (top) and Thr172 phosphorylation (bottom) by LKB1 of AMPK-γ1 complexes expressed in HeLa cells. FLAG-tagged AMPK-γ1, either wild-type (WT) or R299G mutant (RG), was expressed in HeLa cells and AMPK complexes precipitated using anti-FLAG antibody. The complexes (which have low basal phosphorylation due to lack of LKB1 in HeLa cells) were incubated with or without a limiting amount of LKB1, AMP (200 μM) or SU6656 (3 μM) for 10 min and AMPK activity determined. Results are mean ± SEM (n = 6); asterisks and daggers indicate significant differences from controls without LKB1 and without AMP/SU6656, respectively. ***p < 0.001, ****p < 0.0001, ^††††^p < 0.0001; ns, not significant. The lower panel shows western blots of single incubations.

Since SU6656 activation did not require functional γ-subunit or ADaM sites, this left the catalytic site as the most likely binding site. Indeed, SU6656 inhibits AMPK as effectively as Src (Bain et al., 2007). To examine this in more detail, we initially used a purified preparation of rat liver AMPK (Hawley et al., 1996) and conducted assays at 2 mM ATP, when AMP causes a substantial allosteric activation (>5-fold) (Gowans et al., 2013). Under these conditions, SU6656 inhibited both basal and AMP-stimulated activity at concentrations above 1 μM, suggesting that it bound at the catalytic site rather than the γ subunit (Figure 5A). We then switched to the isolated α2 kinase domain (α2-KD, residues 1–310) expressed in bacteria and phosphorylated using CaMKK2. As expected, inhibition of the α2-KD by SU6656 was competitive with ATP, since the data gave good fits to an equation for competitive inhibition (Figure 5B) with Vmax = 141 ± 1 nmol/min/mg, Km_ATP_ = 151 ± 7 μM, and Ki_SU6656_ = 0.17 ± 1 μM.

In a crystal structure of the human α2β1γ1 complex (Xiao et al., 2013), the side chain of Ala156 in α2-KD interacts with the kinase inhibitor staurosporine in the catalytic site, while in a structure of CaMKIIδ with bound SU6656 (Rellos et al., 2010), the equivalent residue interacts with the sulfonamide group of SU6656. We therefore expressed the α2-KD as the WT or an A156T (AT) mutant and phosphorylated both using CaMKK2. Figure 5C shows that WT α2-KD was inhibited by SU6656 (IC_50_ = 0.22 ± 0.02 μM), while inhibition was almost 10-fold less potent with the AT mutant (IC_50_ = 1.7 ± 0.3 μM).

We next used unphosphorylated α2-KD and tested the effect of SU6656 on its activation and phosphorylation by LKB1. Concentrations of SU6656 up to 10 μM promoted activation of WT α2-KD by LKB1 by 1.9 ± 0.2-fold and increased Thr172 phosphorylation (Figures 5D and 5E), while both effects were almost abolished by the AT mutation. There was no effect of SU6656 when LKB1 was assayed using an alternative peptide substrate (Figure S3), confirming that it acts by binding to AMPK rather than to LKB1. The half-maximal effect of SU6656 on α2-KD activation was at 0.20 ± 0.02 μM, close to the IC_50_ for inhibition of catalytic activity (0.22 ± 0.02 μM).

To test whether SU6656 binding inhibited Thr172 dephosphorylation, we used bacterially expressed α2-KD or a full-length α2β2γ1 complex, both of which had been phosphorylated using CaMKK2. Figure 5F shows that protein phosphatase PP2Cα caused inactivation of both constructs, and that this was abolished by the presence of AMP using the α2β2γ1 complex, but not the α2-KD, as expected. However, concentrations of SU6656 up to 10 μM did not protect against inactivation using either construct.

We also expressed FLAG-tagged AMPK-γ1 in HeLa cells, either the WT or an R299G (RG) mutation (equivalent to R531G in AMPK-γ2), which prevents AMP binding at the critical site 3 (Jensen et al., 2015). The AMPK complexes generated were precipitated with anti-FLAG antibody and used as substrates for LKB1 in cell-free assays. Both AMP and SU6656 promoted activation and increased Thr172 phosphorylation by LKB1, using complexes containing WT γ1. However, while the effects of AMP were abolished by the RG mutation, the effects of SU6656 were preserved (Figure 5G). Note that α1 is slightly larger than α2 in humans (64.0 versus 62.3 kDa), and the two isoforms can be resolved by SDS-PAGE.

### SU6656 and AMP Promote Thr172 Phosphorylation by Binding to the Catalytic Site: Studies in Intact Cells

**Figure 6:**
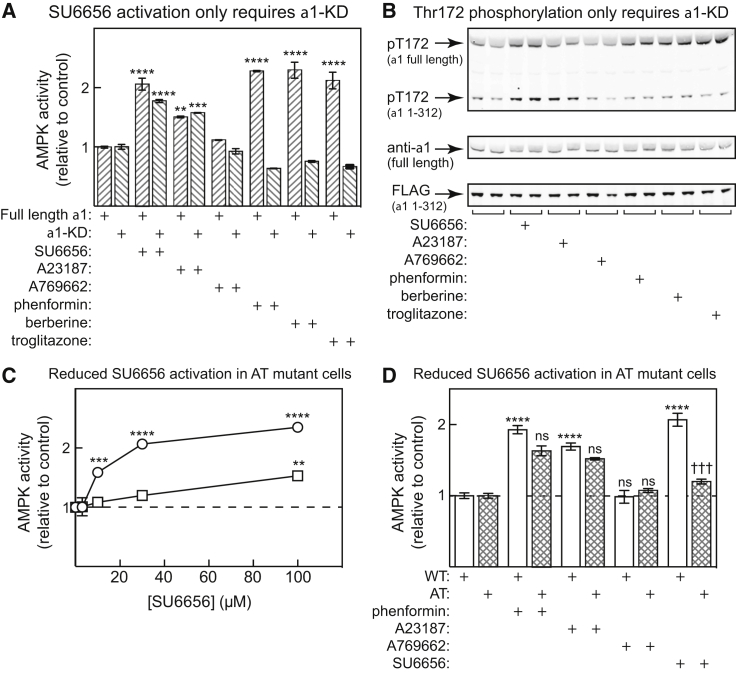
SU6656 Promotes AMPK Activation by Binding to the Catalytic Site: Studies in Intact Cells (A) FLAG-tagged α1 kinase domain (α1-KD, 1–312) was expressed by transient transfection in HEK293 cells and the cells treated with SU6656 (100 μM), A23817 (10 μM), A769662 (300 μM), phenformin (10 mM), berberine (100 μM), or troglitazone (100 μM) for 1 hr. The α1-KD was precipitated using anti-FLAG and endogenous AMPK complexes using anti-α1 antibody, and activity determined. Results (mean ± SD, n = 2) are expressed relative to activities in DMSO controls. (B) Western blots (n = 2) using the indicated antibodies; samples from experiment in (A). (C) DNAs encoding FLAG-tagged AMPK-α1 (WT or AT mutant) were expressed by transient transfection in HEK293 cells, the cells treated with increasing concentrations of SU6656 for 1 hr, AMPK complexes precipitated with anti-FLAG, and activity measured. Results (mean ± SD, n = 3) are expressed relative to the DMSO control. The absolute activities of the WT and AT mutant were 0.14 ± 0.01 (WT) and 0.12 ± 0.01 nmol/min/mg (mean ± SEM, n = 3); in separate experiments we showed that the AT mutant was stimulated 2.6-fold by 200 μM AMP, similar to the WT. (D) As (C), but cells were treated with phenformin (10 mM), A23187 (10 μM), A769662 (300 μM), or SU6656 (100 μM) for 1 hr. Results (mean ± SD, n = 3) are expressed relative to the DMSO control. **p < 0.01, ***p < 0.001, ****p < 0.0001, ^†††^p < 0.001; ns, not significant.

We next expressed an FLAG-tagged α1 kinase domain (α1-KD, residues 1–312) by transient transfection in HEK293 cells, treated the cells with various AMPK activators, and then assayed both the expressed α1-KD (precipitated using anti-FLAG) and endogenous full-length α1 complexes (precipitated using anti-α1 antibodies recognizing an epitope outside the α1-KD). SU6656 increased kinase activity (Figure 6A) and Thr172 phosphorylation (Figure 6B) with both the full-length endogenous α1 complexes and the expressed α1-KD. This was also observed with A23187, as expected since this ionophore increases intracellular Ca^2+^ and activates the alternate upstream kinase CaMKK2. By contrast, three activators that work by increasing cellular AMP/ADP (phenformin, berberine, and troglitazone) all failed to activate or cause Thr172 phosphorylation of the α1-KD while doing this with the endogenous complexes, consistent with the fact that only the latter have AMP/ADP binding sites on the γ subunit. A769662 is primarily an allosteric activator (Goransson et al., 2007, Scott et al., 2014a) and only has modest effects on Thr172 phosphorylation, particularly with γ1 complexes (Ross et al., 2016a). Although there may have been a small effect of A769662 on the activity of immunoprecipitated full-length α1 complexes in Figure 6A, it was not statistically significant.

These results showed that only the kinase domain was required for SU6656 to activate AMPK in intact cells. To confirm that it acted by binding at the catalytic site, we expressed FLAG-tagged full-length AMPK-α1 either as the WT or the AT mutant. Figure 6C shows that the WT was robustly activated by SU6656 up to 100 μM as before, but that activation was greatly reduced with the AT mutant, although there was a small effect at the highest concentration. Figure 6D shows that the WT and AT mutant were activated equally well by the complex I inhibitor phenformin and A23187; once again, A769662 did not cause a significant activation in this experiment. However, activation by SU6656 was significantly reduced by the AT mutation.

### Another AMPK Inhibitor, MRT199665, Also Causes Paradoxical Activation in Cells

**Figure 7:**
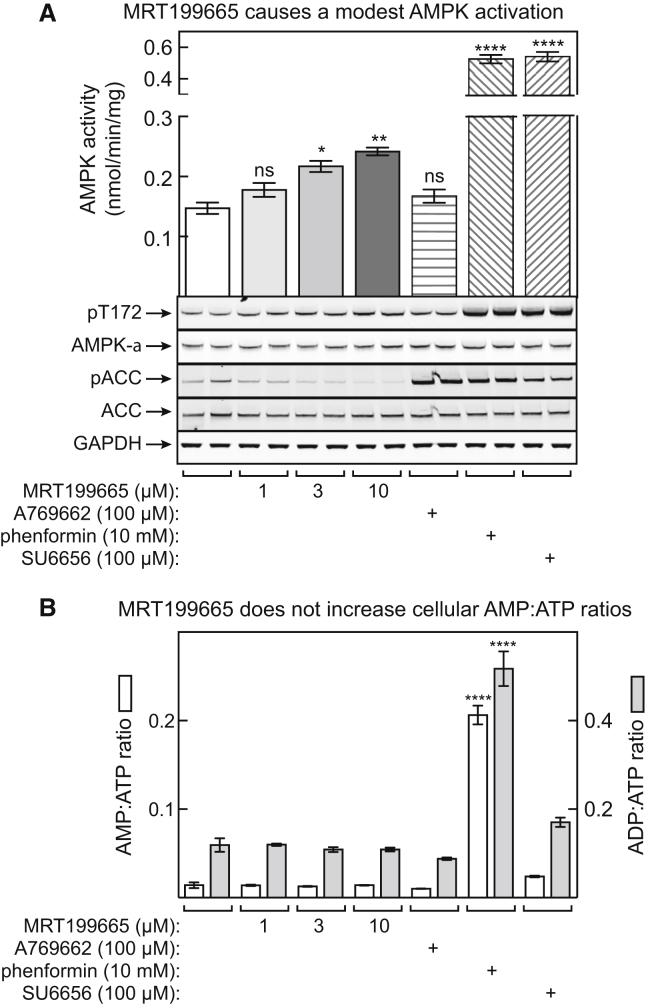
Another AMPK Inhibitor, MRT199665, Activates AMPK but Inhibits ACC Phosphorylation in HEK293 Cells (A) Effect of MRT199665, phenformin (10 mM), A769662 (300 μM), or SU6656 (100 μM) on AMPK activity in HEK293 cells. Cells were incubated for 60 min and AMPK activity measured; results in the top panel are means ± SEM (n = 8), with significant differences from the DMSO control indicated (ns, not significant). The bottom panels show western blots from the same experiment (n = 2). (B) Analysis of AMP:ATP and ADP:ATP ratios from a parallel experiment to that in (A). Results are means ± SEM (n = 3), with significant differences from the DMSO control indicated. *p < 0.05, **p < 0.01, ****p < 0.0001.

We wondered whether other AMPK inhibitors might have the same paradoxical effect as SU6656. However, many known inhibitors, including staurosporine and compound C, inhibit LKB1 with similar potency to AMPK (see MRC Kinase Inhibitor Database, www.kinase-screen.mrc.ac.uk/kinase-inhibitors), so increased Thr172 phosphorylation by LKB1 in intact cells is not observed. By searching this database, we found one other inhibitor, MRT199665, where inhibition of LKB1 was less potent than that of AMPK, as with SU6656. When HEK293 cells were incubated with increasing concentrations of MRT199665, we observed a modest increase in AMPK activity and Thr172 phosphorylation, more than with A769662 but much less than with phenformin or SU6656. However, unlike SU6656, A769662, or phenformin, MRT199665 led to inhibition of phosphorylation of the downstream target ACC (Figure 7A). Activation of AMPK by MRT199665 was not associated with changes in AMP:ATP or ADP:ATP ratios (Figure 7B), so we believe that it was activating AMPK via the same mechanism as SU6656.

## Discussion

Our results show that SU6656 activates AMPK but not by inhibition of phosphorylation of either LKB1 or AMPK by Fyn or another Src family kinase as previously suggested (Yamada et al., 2010, Yamada et al., 2016). The key evidence is: (1) SU6656 did not activate any of the ARKs that are also LKB1-dependent; (2) the effect was not mimicked by another Src kinase inhibitor, PP2; (3) it was still observed in SYF cells, which do not express any Src family kinase; (4) it was still observed in HeLa cells expressing LKB1 with all three tyrosine residues phosphorylated by Fyn and/or Lck mutated; (5) it was still observed in cells expressing AMPK-α2 with a mutation (Y436F) in the tyrosine residue proposed to be phosphorylated by Fyn (Yamada et al., 2016); (6) it was still observed, both in cell-free assays and intact cells, with AMPK-α1/-α2 constructs containing only the kinase domains and therefore lacking Tyr432/436. Although our results cast doubt on these previously proposed mechanisms, they do not invalidate the results showing that SU6656 activated AMPK in mice in vivo, and that this led to increases in fat oxidation and decreases in fat mass (Yamada et al., 2010), which can be explained by SU6656-mediated phosphorylation of ACC by AMPK. In that study, the decrease in respiratory quotient induced by SU6656 (indicating increased fat oxidation) was reported to be absent in Fyn^−/−^ mice. However, the respiratory quotient was already markedly lower in untreated Fyn^−/−^ mice than in WT mice, suggesting that the Fyn knockout may have already increased fatty acid oxidation by some other mechanism, so that any further stimulation by SU6656 might no longer be evident. The authors do not appear to have tested whether SU6656 still activated AMPK in the Fyn knockout mice.

We initially suspected that SU6656 might activate AMPK indirectly by interfering with cellular metabolism and increasing cellular AMP or ADP, since many xenobiotics act by that mechanism (Hawley et al., 2010). Our results rule this out because, at concentrations that caused robust AMPK activation, SU6656: (1) did not increase cellular AMP:ATP/ADP:ATP ratios; (2) did not affect cellular oxygen uptake; (3) still activated AMPK in cells expressing an AMP/ADP-insensitive mutant of the AMPK-γ2 subunit; (4) still promoted activation and phosphorylation by LKB1 in cell-free assays of AMPK complexes containing an AMP/ADP-insensitive mutant of AMPK-γ1. This contrasts with results obtained using another kinase inhibitor, sorafenib, which increased cellular AMP:ATP and ADP:ATP ratios, inhibited cellular oxygen uptake, and failed to activate AMPK in cells expressing the AMP/ADP-insensitive mutant, all at therapeutically relevant concentrations. These results show that sorafenib, which is in clinical use (Gadaleta-Caldarola et al., 2015), activates AMPK indirectly by inhibiting mitochondrial metabolism, a mechanism similar to that of metformin.

By studying the effects of mutations in the ADaM site, we also ruled out that SU6656 activates AMPK by binding to that site. We did confirm that MT 63-78 activates AMPK by binding the ADaM site, which had been suspected (Zadra et al., 2014) but not directly demonstrated. One surprising finding was that mutations at the ADaM site not only blocked the effect of A769662 in intact cells but also the effects of agents that increase cellular AMP/ADP levels to promote Thr172 phosphorylation, although they did not block allosteric activation by AMP. Thus, the effect of AMP on net Thr172 phosphorylation requires not only intact AMP-binding sites (Hawley et al., 2010) but also a functional ADaM site. These results support previous findings that neither AMP nor A769662 protected Thr172 from dephosphorylation in heterotrimers containing N-terminally truncated β subunits lacking the β-CBM, although allosteric activation by AMP occurred normally (Xiao et al., 2013). Since the β-CBM forms one surface of the ADaM-binding cleft, those constructs would also have lacked an intact ADaM site.

While ruling out these various mechanisms for activation by SU6656, our results point instead to a surprising mechanism in which SU6656 binds to the catalytic site on the α subunit. By competing with ATP, this causes acute inhibition of AMPK, yet paradoxically simultaneously promotes phosphorylation at the activating site, Thr172. Binding at the catalytic site is consistent with studies showing that SU6656 inhibits numerous protein kinases, including AMPK (Bain et al., 2007). In a structure of CaMKIIδ crystallized with the compound, SU6656 was bound in the catalytic site with its adenine-like moiety in the position normally occupied by the adenine of ATP (Rellos et al., 2010). We found that SU6656 inhibited both basal and AMP-stimulated AMPK activity, suggesting that it bound at the catalytic site rather than one of the regulatory AMP-binding sites on the γ subunit. Consistent with this, inhibition was competitive with ATP and was observed in cell-free assays using the isolated α2 kinase domain, but was greatly reduced by an A156T mutation (in the CaMKIIδ structure [Rellos et al., 2010], the equivalent residue interacts with the sulfonamide group of SU6656). Ala156 also lies at the start of the activation loop, whose conformation is important for the binding of many kinase inhibitors; substitution of the equivalent alanine with threonine (A2016T) in the LRRK2 kinase domain greatly reduced the potency of three different inhibitors (Nichols et al., 2009).

Despite the acute inhibitory effect of SU6656 on AMPK kinase activity, its binding appears to cause a conformational change that enhances phosphorylation of Thr172 on the activation loop by LKB1. This was not due to direct binding to LKB1, since LKB1 activity using an alternate peptide substrate was unaffected by SU6656. We did not observe any effect of SU6656 on inactivation of AMPK by protein phosphatases in cell-free assays.

Recently, the biquinoline JJO-1 was reported to activate AMPK (Scott et al., 2014b) and, since it only activated AMPK at low ATP concentration (<100 μM), its effects initially appeared to be competitive with ATP. However, unlike SU6656, JJO-1 did not activate or inhibit an AMPK construct containing only the kinase domain (α1, 1–315), suggesting that it did not bind at the catalytic site. Also, it did not promote Thr172 phosphorylation but acted in a purely allosteric manner by binding at an undefined site. JJO-1 therefore acts via a different mechanism from SU6656.

Other AMPK inhibitors, e.g., compound C, do not cause the same paradoxical activation of AMPK as SU6656, because they also inhibit the upstream kinases LKB1 and CaMKK2. By searching a kinase inhibitor database, we identified another compound, MRT199665, that was a more potent inhibitor of AMPK than of LKB1. Like SU6656, it caused a paradoxical activation and Thr172 phosphorylation of AMPK in cells, although its effects were much more modest. It did not affect cellular AMP:ATP or ADP:ATP ratios, suggesting that it may act via the same mechanism as SU6656. However, unlike SU6656, MRT199665 inhibited rather than increased phosphorylation of the downstream target, ACC, in intact cells.

It might seem paradoxical that compounds that bind in the ATP-binding catalytic site and therefore inhibit the kinase should promote activation by phosphorylation of the activation loop. This is not unprecedented, since catalytic site inhibitors of PKB/Akt promote its phosphorylation, both at Thr308 within the activation loop and Ser473 within the hydrophobic motif (Okuzumi et al., 2009). However, the inhibitors still blocked phosphorylation of downstream targets of Akt in intact cells, whereas in our study SU6656 (although not MRT199665) promoted phosphorylation of at least one downstream target, ACC. We believe this paradoxical activation of AMPK by inhibitors binding in the catalytic site can be explained by assuming that inhibitor binding causes a conformational change in the activation loop that promotes Thr172 phosphorylation. The lifetime of the active, phosphorylated state must then be sufficiently long to allow dissociation of the inhibitor and its replacement by ATP, so that one or more cycles of catalysis can occur before Thr172 dephosphorylation. The overall effect will depend on several factors, such as the turnover rate of phosphorylated Thr172 in the cells, the off-rate of the inhibitor, and the on-rate of the target protein at the catalytic site. The effect might be particularly evident with substrates that bind to AMPK with high affinity such as ACC (Scott et al., 2002), explaining why SU6656 increased the phosphorylation of ACC, but not Raptor, in intact cells (Figure 1A). Our model also explains why SU6656 caused a much larger Thr172 phosphorylation than berberine, yet ACC phosphorylation was comparable. With SU6656, the effect of increased Thr172 phosphorylation would be partly counteracted by inhibition of catalytic activity, whereas with berberine, the increase in cellular AMP would not only enhance net Thr172 phosphorylation but also cause allosteric activation. Thus, the same degree of ACC phosphorylation would be achieved with a much lower degree of Thr172 phosphorylation using berberine rather than SU6656.

## Significance

**Activation of AMPK by SU6656 occurs not by inhibition of tyrosine phosphorylation of LKB1 or AMPK by Src kinases as previously suggested, but by binding of SU6656 at the catalytic site of AMPK. This acutely inhibits the kinase activity, yet paradoxically causes a conformational change that activates the kinase by promoting phosphorylation of Thr172 on the activation loop by LKB1. This causes increased phosphorylation of some, but not all, downstream targets in intact cells. MRT199665 appears to act by a similar mechanism to SU66556, but sorafenib activates AMPK indirectly by inhibiting mitochondrial metabolism and increasing cellular ADP/AMP levels. Nevertheless, this effect of sorafenib occurs at concentrations similar to peak plasma concentrations observed in humans. Our results are significant because there is a current interest in the development of AMPK inhibitors for treatment of cancer; such approaches will need to consider the possibility that the inhibitors might also cause paradoxical activation by one of these two mechanisms.**

## STAR★Methods

### Key Resources Table

**Table:** 

REAGENT or RESOURCE	SOURCE	IDENTIFIER
**Antibodies and Other Probes**		

GAPDH	Sigma-Aldrich	Cat# G9545; RRID: AB_796208
pT172 (AMPK-α)	Cell Signaling Technology	Cat# 2531; RRID: AB_330330
pS108 (AMPK-β1)	Cell Signaling Technology	Cat# 4181; RRID: AB_10841303
pS792 (Raptor)	Cell Signaling Technology	Cat# 2083; RRID: AB_2249475
Raptor	Cell Signaling Technology	Cat# 2280; RRID: AB_10694695
pT202/pY204 (Erk1/Erk2)	Cell Signaling Technology	Cat# 4370; RRID: AB_2315112
Erk1/Erk2	Cell Signaling Technology	Cat# 9107; RRID: AB_10695739
GFP	Cell Signaling Technology	Cat# 2956; RRID: AB_1196615
pACC1/pACC2 (S79/S212)	Cell Signaling Technology	Cat# 11818
AMPK, pan-α (for Western blots)	Cell Signaling Technology	Cat# 2532; RRID: AB_330331
AMPK-α1 (for immunoprecipitation)	(Woods et al., 1996)	N/A
AMPK-α2 (for immunoprecipitation)	(Woods et al., 1996)	N/A
AMPK-β1	(Cheung et al., 2000)	N/A
AMPK-β2	(Cheung et al., 2000)	N/A
SIK1	(Lizcano et al., 2004)	N/A
SIK2	(Lizcano et al., 2004)	N/A
SIK3	(Lizcano et al., 2004)	N/A
SNARK	(Lizcano et al., 2004)	N/A
MARK2/3	(Lizcano et al., 2004)	N/A
MARK4	(Lizcano et al., 2004)	N/A
EZview Red ANTI-FLAG M2 Affinity Gel	Sigma-Aldrich	Cat# F2426; RRID: AB_2616449
Streptavidin DyLight™ 800 Conjugated	Rockland	Cat# S000-45

**Biological Samples**		

AMPK, purified from rat liver	(Hawley et al., 1996)	N/A

**Chemicals, Peptides, and Recombinant Proteins**		

SU6656	Sigma-Aldrich	Cat# S9692
sorafenib	Santa-Cruz	Cat# SC-220125A
berberine chloride	Sigma-Aldrich	Cat# B3251
phenformin hydrochloride	Sigma-Aldrich	Cat# P7045
PP2	Sigma-Aldrich	Cat# P0042
troglitazone	Tocris-Bioscience	Cat# 3114
A769662	(Goransson et al., 2007)	N/A
MRT199665	(Clark et al., 2012)	N/A
FuGENE® 6 transfection reagent	Promega	Cat# E2691
PP2Cα *(PPM1A)*	(Davies et al., 1995)	N/A
GST-LKB1 *(STK11)*	(Boudeau et al., 2003)	N/A
FLAG-tagged STRAD-α *(STRADA)*	(Boudeau et al., 2003)	N/A
Myc-tagged MO25-α *(CAB39)*	(Boudeau et al., 2003)	N/A
GFP-tagged LKB1 *(STK11)*	(Fogarty and Hardie, 2009)	N/A
Human AMPK (α1β1γ1 complex) *(PRKAA1:PRKAB1:PRKAG1)*	generated as in (Neumann et al., 2003); gift from Mark Peggie, University of Dundee	N/A
Human AMPK (α2β2γ1 complex) *(PRKAA2:PRKAB2:PRKAG1)*	generated as in (Neumann et al., 2003); gift from AstraZeneca	N/A
Human LKB1 purified from Sf9 cells (LKB1: STRADα: MO25α complex) *(STK11:STRADA:CAB39)*	generated as in (Jaleel et al., 2006); gift from Division of Signal Transduction, University of Dundee	N/A
Brij-35 (30% solution)	BDH (now VWR)	Cat# 224175X
phenylmethane sulphonyl fluoride	Sigma-Aldrich	Cat# P7626
benzamidine	Sigma-Aldrich	Cat# B6506
soybean trypsin inhibitor	Sigma-Aldrich	Cat# T9128
bovine serum albumin	Sigma-Aldrich	Cat# A7906

**Experimental Models: Cell Lines**		

SYF	ATCC	Cat# CRL-2459
HEK-293	ECACC	Cat# 85120602
Flp-In™ T-REx™ 293	ThermoFisher Scientific	Cat# R78007
HeLa	ECACC	Cat# 93021013

**Recombinant DNA**		

Plasmid encoding full-length human AMPK-α1	(Hawley et al., 2014)	N/A
Plasmid encoding full-length human AMPK-γ1	(Jensen et al., 2015)	N/A
Plasmid encoding AMPK-α1 kinase domain (α1-KD)	this paper	N/A
Plasmid encoding AMPK-α2 kinase domain (α2-KD)	this paper	N/A

### Contact for Reagent and Resource Sharing

Further information and requests for resources and reagents should be directed to and will be fulfilled by the Lead Contact, Grahame Hardie (d.g.hardie@dundee.ac.uk).

### Experimental Model and Subject Details

#### Cell Lines

SYF cells were from the ATCC and were used within 3 months of purchase. Human cells (HEK-293 and HeLa) were from the European Collection of Cell Cultures (ECACC) and were re-validated by STR profiling by Public Health England prior to the project (certificate dated 08/14/2015). All cells were cultured in Dulbecco’s Modified Eagle’s Medium (DMEM) with 10% (v/v) FBS and 1% (v/v) penicillin/streptomycin.

### Method Details

#### DNA Plasmids and Site-Directed Mutagenesis

A plasmid encoding the AMPK-α1 kinase domain (α1-KD) was made by amplifying residues 1-312 with an N-terminal FLAG-tag and 5' *Kpn1* and 3' *Xho1* sites, the resulting PCR product was cloned into pcDNA5 FRT. A plasmid encoding the AMPK-α2 kinase domain (α2-KD) was made by amplifying residues 1-310 with 5' *BamH1* and 3' *Xho1* sites, the resulting PCR product was cloned into pGEX6P2. Point mutations were generated using the Quikchange II site-directed mutagenesis kit (Stratagene) and confirmed by DNA sequencing.

#### Stable Cell Lines and Transient Transfection

HEK-293 cells stably expressing tetracycline-inducible FLAG-tagged human AMPK-γ2, or an R531G mutant, were generated by introducing the corresponding DNAs into Flp-In™ T-REx™ 293 cells (Auciello et al., 2014). Expression of the FLAG-tagged constructs was induced for 48 hr using tetracycline (1 μg.ml^-1^) prior to addition of compounds under test. Transient transfections were carried out 36-48 hr prior to experiments, using Fugene 6 according to manufacturers’ instructions.

#### Analysis of Commercial SU6656

Semi-preparative HPLC (see Figure S5) was performed on a Buchi PrepChrom C-700 LC system with a 250 x 21.2 mm (ID) preparative column (PrepChrom C18, 10 μm particle size, Buchi) at a flow rate of 25 mL/min. A linear gradient from 5%-95% acetonitrile in water over 18 min was used for elution. Detection was at 254 nm and via a dual scan at 220/400 nm.

NMR spectra were recorded on Bruker AVANCE II 500 spectrometer. Splitting patterns of spectral multiplets are indicated as s, singlet; br s, broad singlet; d, doublet; dd doublet of doublets; m, multiplet. Analysis of SU 6656 supplied by Sigma: ^1^H NMR (500 MHz, d6-DMSO) δ 13.15 (br s, 1H), 11.30 (s, 1H), 8.05 (d, *J* 1.5 Hz, 1H), 8.0 (s, 1H), 7.54 (dd, *J* 8.0Hz, *J* 1.6 Hz, 1H), 7.13 (d, *J* 8.0 Hz, 1H), 6.76 (br s, 1H), 2.80 (m, 2H), 2.66 (s, 6H), 2.60 (m, 2H), 1.86 (m, 2H), 1.78(m, 2H). Material purified by semi-preparative HPLC gave an identical spectrum.

LC-MS analysis was performed using a Dionex UltiMate3000 series LC instrument with a Waters Xbridge C18 column (3.5 μm particle size, 2.1 mm x 50 mm) at a flow rate 0.3 mL/min. A linear gradient from 5%-95 % buffer B in buffer A over 8 min was used for elution (buffer A: water/0.1% formic acid; buffer B: acetonitrile/0.1% formic acid). The LC was connected to a Bruker MicroTOF and MS was acquired in ESI positive mode. The major peak detected by UV absorbance at 254 nm generated a m/z value ([M+H]^+^) of 372.4 (expected mass for C_19_H_21_N_3_O_3_S = 371.46), as well as a [2M+H]^+^ peak of 743.3 (not shown).

Samples of the preparation of SU6656 received from the supplier, and that purified by semi-preparative HPLC as described above, were dried down, dissolved in DMSO and tested for their ability to activate and trigger Thr172 phosphorylation of AMPK in HEK-293 cells. The results for both preparations were indistinguishable, once again confirming the purity of the commercial preparation (Figure S6).

#### Synthesis and Analysis of MT 63-78

MT 63-78 was synthesized as described previously (Birnberg et al., 2009). LC:MS was performed on an Agilent 1100 series using a Waters Xbridge C18 column (3.5 μm particle size, 2.1 mm x 50 mm, flow rate 0.3 mL/min, linear gradient 5%-95 % buffer B in buffer A in 7 min; buffer A: water-0.1% ammonia; buffer B: acetonitrile-0.1% ammonia); MS was by a Bruker MicrOTOF mass spectrometer. LCMS ESI (+): R_t_ = 3.7 min; [M+H]^+^= 327.12, expected for C_21_H_15_N_2_O_5_, 327.11. NMR spectra were recorded on Bruker AVANCE II 500 spectrometer. Splitting patterns of spectral multiplets are indicated as s, singlet; d, doublet; m, multiplet: ^1^H NMR (500 MHz, CD_3_OD) δ 8.28 (s, 1H), 8.15 (s, 1H), 8.08 (d, 2H), 7.95-7.84 (m, 4H), 7.39-7.36 (m, 1H), 6.86 (d, 2H); ^13^C NMR (126 MHz, CD_3_OD) δ 155.3, 140.5, 135.7, 135.2, 133.8, 133.0, 131.6, 128.8, 128.1, 127.5, 123.7, 117.4, 116.8, 116.5, 113.1, 107.4, 85.9.

#### Kinase Assays

Endogenous AMPK was immunoprecipitated using the specified antibodies (for total AMPK, an equal mixture of anti-α1 and –α2 antibodies) and assayed at 200 μM ATP (Hardie et al., 2000, Towler et al., 2008) using the *AMARA* peptide (Dale et al., 1995) as substrate, except for assays of allosteric activation by AMP (Figure 5A) where the *SAMS* peptide was used as substrate with 2 mM ATP (Gowans et al., 2013). The AMPK-related kinases SIK1, SIK2, SIK3, SNARK, MARK2/3 and MARK4 were immunoprecipitated and assayed as previously described (Lizcano et al., 2004) in lysates of Hela cells stably expressing LKB1 (Fogarty et al., 2010). LKB1 was assayed using the LKBtide peptide as substrate (Sakamoto et al., 2004).

#### Assays of Phosphorylation/Dephosphorylation

##### Phosphorylation

Plasmids encoding FLAG-tagged AMPK- γ1 (WT or RG mutant) were transfected into HeLa cells using Fugene 6. Cell lysate (90 μg protein) was immunoprecitated using EZview Red anti-FLAG M2 affinity gel, and incubated for 10 min in a total volume of 75 μl with 200 μM ATP, 5 mM MgCl_2_ ± a limiting amount of LKB1:STRAD:MO25 complex (0.7 μg), in the presence or absence of AMP (200 μM) or SU6656 (3 μM) as indicated. The reaction was terminated by addition of 1 ml of ice-cold IP buffer (50 mM Tris/HCl, pH 7.4 at room temp, 150 mM NaCl, 1 mM EGTA, 1 mM EDTA, 5 mM Na pyrophosphate, 50 mM NaF, 1 mM dithiothreitol, 0.1% (v/v) Triton X-100, 0.1 mM phenylmethane sulphonyl fluoride, 5 μg/ml soybean trypsin inhibitor, 1 mM benzamidine, and after an additional wash in the same buffer the AMPK activity or Thr172 phosphorylation status was determined by kinase assay or Western blotting.

##### Dephosphorylation

Active human AMPK (α1β1γ1 complex) phosphorylated on Thr172 was generated as described previously (Hawley et al., 2012). Dephosphorylation assays contained AMPK, 2 mM AMP or various concentrations of SU6656, 5 mM ATP, 1 mg/ml bovine serum albumin and 60 μg/ml PP2Cα in Hepes buffer (50 mM Na Hepes, pH 7.4, 150 mM NaCl, 1 mM dithiothreitol, 0.02% (w/v) Brij-35). The reaction was started by adding MgCl_2_ (9.8 mM) to activate PP2Cα; reactions were conducted in a shaking incubator at 30°C for 10 min and were stopped by diluting 5 μl into 300 μl Hepes buffer (as above). An aliquot (5 μl) was then taken to assay AMPK.

#### Measurement of AMP:ADP and ADP:ATP Ratios

The levels of AMP, ADP and ATP were measured using a TSQ Quantiva interfaced with Ultimate 3000 Liquid Chromatography system (ThermoScientific), equipped with a porous graphitic carbon column (HyperCarb 30x1mm ID 3 μm; Part No: C-35003-031030, Thermo-Scientific). Mobile phase buffer A consisted of 0.3% (v/v) formic acid adjusted to pH 9 with ammonia prior to a 1:10 dilution. Mobile phase buffer B was 80% (v/v) acetonitrile. The column was maintained at a controlled temperature of 30°C and was equilibrated with 10% buffer B for 5 minutes at a constant flow rate of 0.06 mL/min. Aliquots of 1 μL of each sample were loaded onto the column and compounds eluted with a linear gradient of 10%-60% buffer B over 9 min. Buffer B was then increased to 100% within 1 min, and the column washed for 5 min with 100% Buffer B. Eluents were sprayed into the TSQ Quantiva using Ion Max NG ion source with ion transfer tube temperature set to 350°C and vaporizer temperature 125°C. The TSQ Quantiva was run in negative mode with a spray voltage of 2600, sheath gas 40 and Aux gas 10. Levels of ATP, ADP and AMP were measured using multiple reactions monitoring mode (MRM) with optimised collision energies and radio frequencies previously determined by infusing pure compounds. Three transitions were used to monitor each of the three compounds, ATP (505.92>158.98, 505.92>408.12 and 505.92>426.12), ADP (426.98>158.98, 426.98>328.78 and 426.98>409.09) and AMP (345.96>134.20, 345.96>151.27 and 345.96>211.12).

#### Measurements of Cellular Oxygen Consumption

Cellular oxygen consumption rate was measured using a Seahorse XF24 Extracellular Flux Analyser according to manufacturers’ instructions, as specified previously (Hawley et al., 2010).

#### Curve Fitting

Data were fitted to the specified equations by non-linear regression using GraphPad Prism 6 for Mac OSX.

#### Other Analytical Procedures

SDS-PAGE was performed using precast Bis-Tris 4–12% gradient polyacrylamide gels in the MOPS buffer system (ThermoFisher Scientific). Proteins were transferred to nitrocellulose membranes using the iBlot 2 system (ThermoFisher Scientific). Membranes were blocked for 1 hr in Li-Cor Odyssey blocking buffer. The membranes were probed with appropriate antibody (0.1–1 μg/ml) in TBS-Tween and 2% (w/v) non-fat dried skimmed milk except where the blotting enhancement system was used (as per manufacturers’ instructions). Detection was performed using secondary antibody (1 μg/ml) coupled to IR 680 or IR 800 dye, and the membranes were scanned using the LICOR Odyssey IR imager. Protein concentrations were determined by Coomassie Blue binding with bovine serum albumin as standard (Bradford, 1976). The ArnA contaminant in the human α1β1γ1 complex expressed in *E. coli* was identified by peptide mass fingerprinting.

### Quantification and Statistical Analysis

Numbers of replicates and statistical significance is indicated in Figures or Figure legends. Numbers of replicates (n) refer to biological replicates, i.e. the number of independent cell cultures analyzed or independent cell-free assays conducted. Significances of differences were estimated with GraphPad Prism 6 for Mac OSX, using 1-way or 2-way ANOVA as appropriate, and (unless stated otherwise) Sidak’s multiple comparison test. Significant differences are indicated either by asterisks: *P<0.05, **P<0.01, ***P<0.001, ****P<0.0001, or by daggers: †P<0.05, ††P<0.01, †††P<0.001, ††††P<0.0001.

## Author Contributions

F.A.R., S.A.H., F.R.A., and G.J.G. designed and conducted experiments; A.A. and D.J.L. devised a new analytic method and conducted analyses; D.G.H. supervised the project and wrote the first draft of the manuscript; all authors read and amended the manuscript.

## Supplemental Information

Document S1. Figures S1–S5

Document S2. Article plus Supplemental Information
