# Supplementary material for: Mechanisms of Paradoxical Activation of AMPK by the Kinase Inhibitors SU6656 and Sorafenib
Source: Cell Chem Biol. 2017 Jul 20;24(7):813–824.e4. doi: 10.1016/j.chembiol.2017.05.021 (PMC5522529; doi:10.1016/j.chembiol.2017.05.021)
Supplement: Document S1. Figures S1–S5 [file mmc1.pdf]

Cell Chemical Biology, Volume *24*

Supplemental Information

Mechanisms of Paradoxical Activation

of AMPK by the Kinase

Inhibitors SU6656 and Sorafenib

Fiona A. Ross, Simon A. Hawley, F. Romana Auciello, Graeme J. Gowans, Abdelmadjid Atrih, Douglas J. Lamont, and D. Grahame Hardie

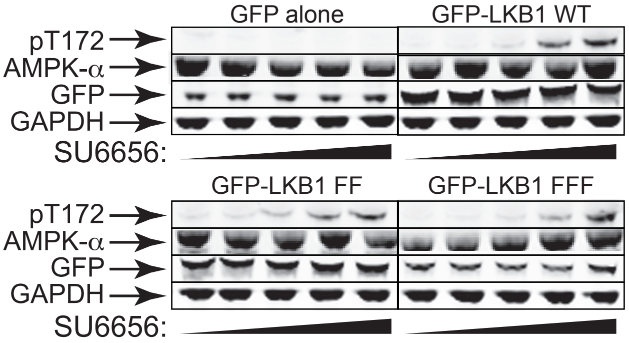

Fig. S1 (related to Fig. 2C): Increased Thr172 phosphorylation in response to SU6656 is independent of phosphorylation of LKB1 at Tyr36, Tyr261 or Tyr365. The results are from the same experiment as Fig. 2C, but show blotting for Thr172, AMPK-α, GFP and GAPDH (loading control).

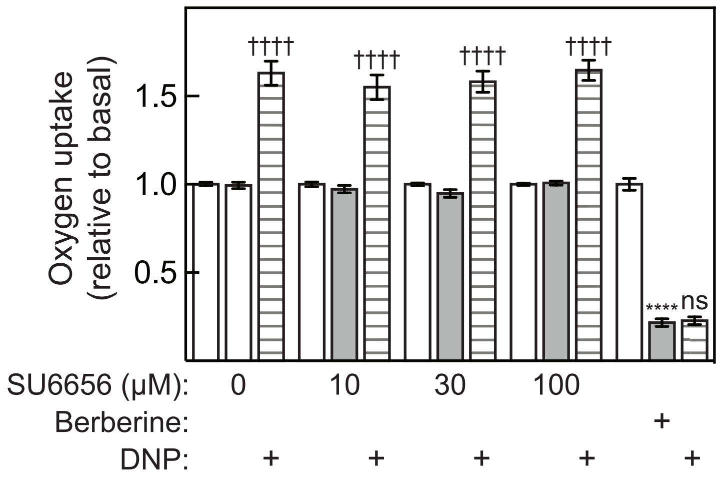

Fig. S2 (related to Fig. 3E): SU6656 does not affect cellular oxygen uptake.
Oxygen uptake of HEK-293 cells measured under basal conditions (open bars), after addition of the indicated concentration of SU6656 or berberine (300 μM) (gray bars), then after subsequent addition of the uncoupler 2,4-dinitrophenol (DNP, hatched bars, 100 μM) (mean ± SEM, n = 6-9). Asterisks denote results significantly different before and after addition of SU6656 or berberine, while daggers denote results significantly different before and after subsequent addition of DNP.

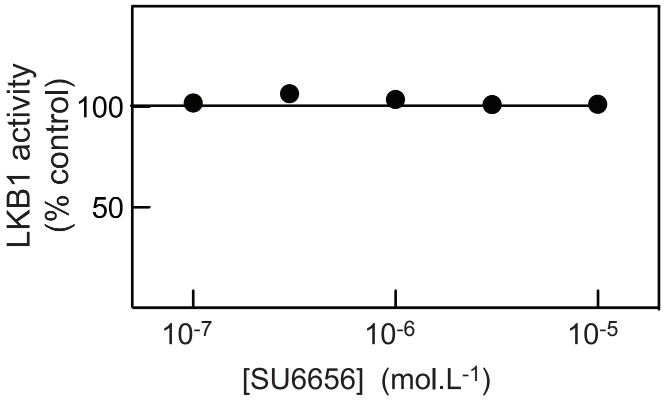

Fig. S3 (related to Fig. 5D/E): SU6656 does not directly affect the kinase activity of LKB1. LKB1 was assayed using the LKBtide peptide as substrate as described in the Star Methods section.

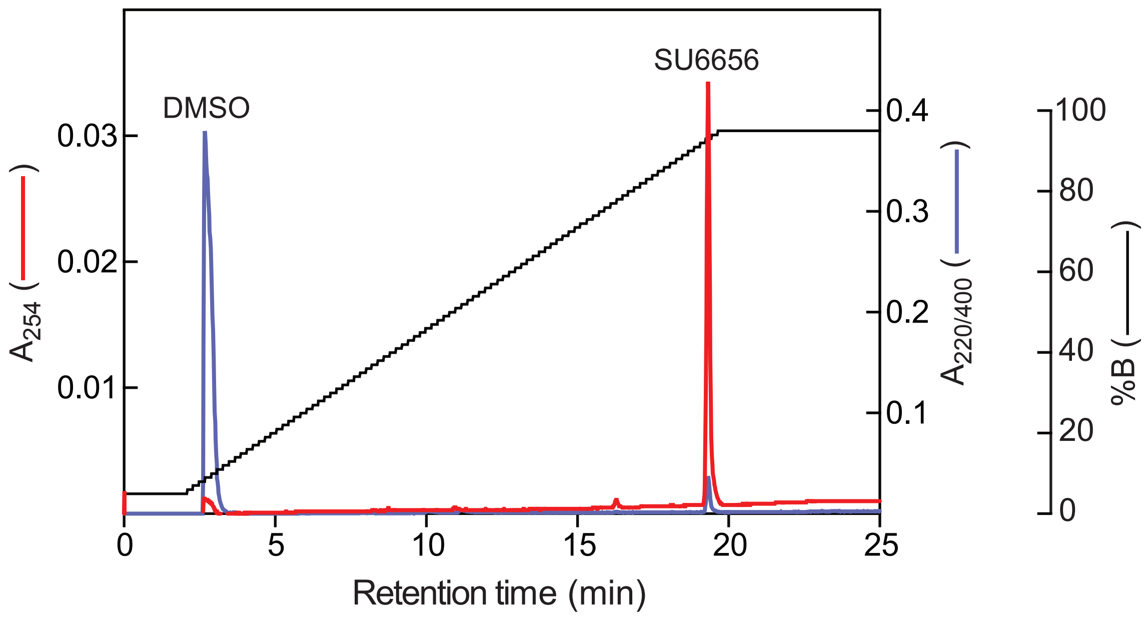

Fig. S4 (related to Star Methods): Semi-preparative HPLC of commercial SU6656. The red trace is absorbance at 254 nm, the blue trace is the signal obtained using a dual wavelength scan (220/400 nm) and the black trace is the % buffer B in the eluent. The peak eluting at 19.5 min was confirmed to be SU6656 by NMR spectroscopy.

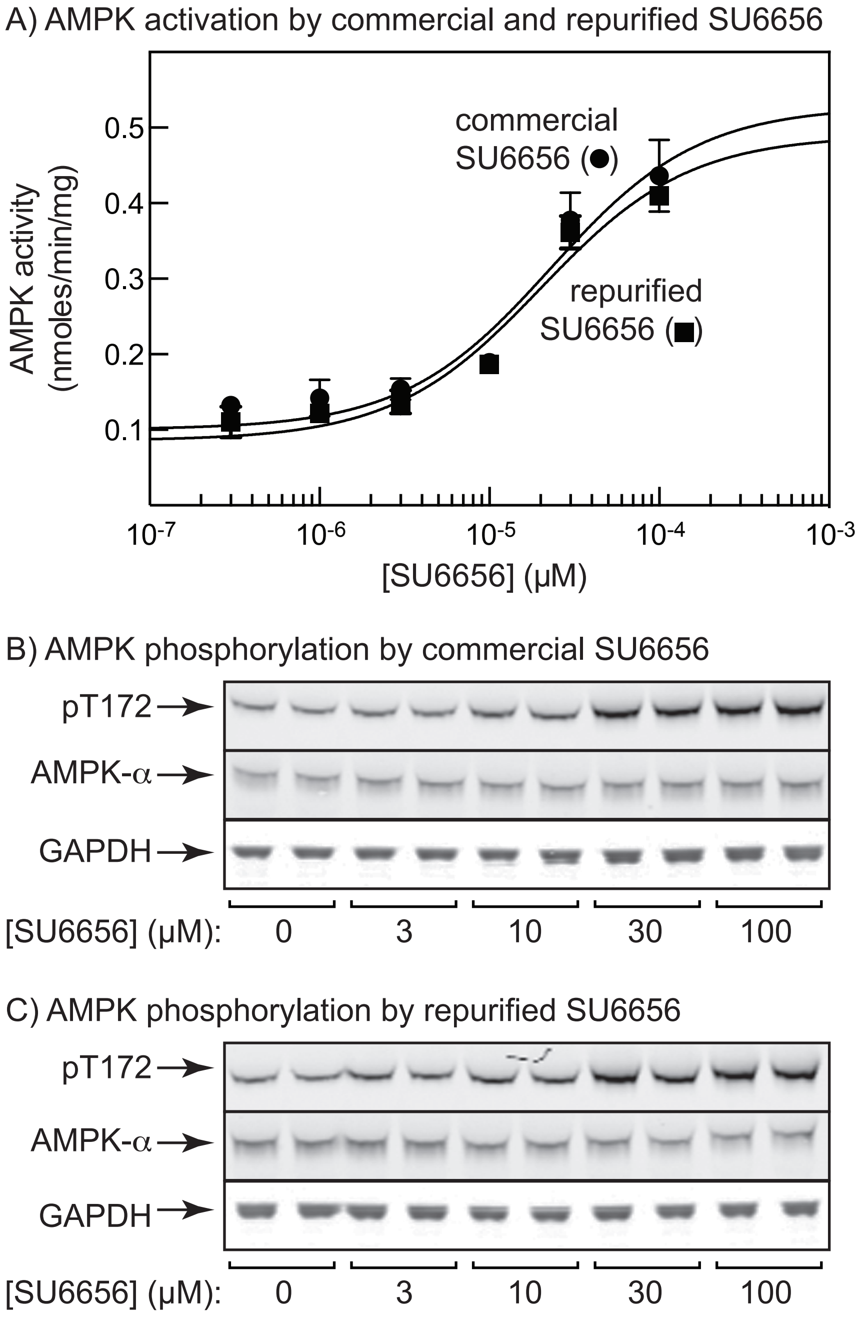

Fig. S5 (related to Star Methods): Activation (A) and phosphorylation (B, C) of AMPK by SU6656 is identical for commercial SU6656 and material repurified by HPLC. SU6656 was used either direct from the supplier or was repurified by semi-preparative HPLC as shown in Fig. S4. The concentration of the repurified material was confirmed by comparing the absorbance at 260 nm with that of the commercial material. HEK-293 cells were then incubated with unpurified or repurified SU6656 and activation and phosphorylation of AMPK determined as in Fig. 1A.
